# Sulfatases: Critical Enzymes for Algal Polysaccharide Processing

**DOI:** 10.3389/fpls.2022.837636

**Published:** 2022-04-28

**Authors:** Andrew G. Hettle, Chelsea J. Vickers, Alisdair B. Boraston

**Affiliations:** ^1^Department of Biochemistry and Microbiology, University of Victoria, Victoria, BC, Canada; ^2^School of Biological Sciences, Victoria University of Wellington, Wellington, New Zealand

**Keywords:** sulfatase, polysaccharide, marine, structure, bacteria

## Abstract

Microbial sulfatases are important biocatalysts in the marine environment where they play a key role in the catabolic biotransformation of abundant sulphated algal polysaccharides. The sulphate esters decorating algal polysaccharides, such as carrageenan, fucoidan and ulvan, can constitute up to 40% of the biopolymer dry weight. The use of this plentiful carbon and energy source by heterotrophic microbes is enabled in part by the sulfatases encoded in their genomes. Sulfatase catalysed hydrolytic removal of sulphate esters is a key reaction at various stages of the enzymatic cascade that depolymerises sulphated polysaccharides into monosaccharides that can enter energy yielding metabolic pathways. As the critical roles of sulfatases in the metabolism of sulphated polysaccharides from marine algae is increasingly revealed, the structural and functional analysis of these enzymes becomes an important component of understanding these metabolic pathways. The S1 family of formylglycine-dependent sulfatases is the largest and most functionally diverse sulfatase family that is frequently active on polysaccharides. Here, we review this important sulfatase family with emphasis on recent developments in studying the structural and functional relationship between sulfatases and their sulphated algal polysaccharide substrates. This analysis utilises the recently proposed active site nomenclature for sulfatases. We will highlight the key role of sulfatases, not only in marine carbon cycling, but also as potential biocatalysts for the production of a variety of novel tailor made sulphated oligomers, which are useful products in, for example, pharmaceutical or cosmetic applications.

## Introduction

Sulphated polysaccharides polysaccharides, such as carrageenan, fucoidan and ulvan, are predominately produced by algae as structural components of their cell walls and represent one of the largest and most diverse sinks of organic carbon in the ocean (Arnosti et al., 2021). The sulphate esters are hypothesised to be an adaptation to marine life as these modifications are far less common in land-based plants, yet in the ocean they can constitute up to 40% of the dry weight of these polymers (Anderson et al., 1965; Aquino et al., 2011; Arnosti et al., 2021). Sulphate esters and other anionic modifications of algal polysaccharides impart unique structural and physicochemical properties that are of increasing relevance to multiple industries (Jiao et al., 2011; Vishchuk et al., 2011; Cunha and Grenha, 2016; Wang et al., 2019; Kidgell et al., 2020; Kwon et al., 2020; Silchenko et al., 2021). Carrageenans, for example have been used extensively in the food industry for decades as gelling, stabilising and clarifying agents (Usov, 1998). Application of fucoidans, and more recently ulvans, are also becoming an important source of high value sustainable products relevant to the food and pharmaceutical industry (Wang et al., 2019; Kwon et al., 2020). For example, two anti-viral drug candidates (RPI-27 and RPI-28; Kwon et al., 2020) that effectively inhibit SARS-CoV-2 are both derived from fucoidans. Recent work has highlighted the role that sulfatases play in both natural cycling of algal polysaccharides and in polysaccharide remodelling for industrial applications. As the key role of sulfatases in processing sulphated polysaccharides comes to light, the need increases for more detailed biochemical analysis of these key enzymes.

Bacteria have evolved unique metabolic capacities to utilise algal polysaccharides as a nutrient source. The major known utilizers belong to the Bacteroidetes, the PVC superphylum and Gamma proteobacteria (Akagawa-Matsushita et al., 1992; Hehemann et al., 2010; Thrash et al., 2010; Wegner et al., 2013; Chen et al., 2016; Barbeyron et al., 2016b; Pluvinage et al., 2018; Hettle et al., 2019; Van Vliet et al., 2019; Sichert et al., 2020). Recent work has highlighted the impressive suite of enzymes that these bacteria encode in their genomes (Mann et al., 2013; Kabisch et al., 2014; Reintjes et al., 2017; Unfried et al., 2018; Kappelmann et al., 2019; Francis et al., 2021; Orellana et al., 2021). The encoded metabolic systems provide the capacity to assimilate and metabolise sulphated polysaccharides. An individual bacterial genome can encode hundreds of S1 family sulfatases that function at various stages of polysaccharide depolymerisation (Thrash et al., 2010; Wegner et al., 2013; Van Vliet et al., 2019).

The large variety of sulphated polysaccharide structures implies a corresponding number of sulfatases with different substrate specificities. Despite advances in annotating genomic and metagenomic data of microorganisms, the correct functional annotation of carbohydrate sulfatases is still difficult due to the limited number of biochemically characterised sulfatases that act on algal polysaccharides. To date, 18 marine carbohydrate-specific sulfatases from nine different bacteria have reported activities (Weigl and Yaphe, 1966; Préchoux et al., 2013, 2016; Genicot et al., 2014; Ficko-Blean et al., 2017; Hettle et al., 2018, 2019; Silchenko et al., 2018; Reisky et al., 2019; Wang et al., 2019; Robb et al., 2022; Table 1). Of these characterised sulfatases nine are active on carrageenans, three on ulvan, four on fucoidans and two on porphyran. This is comparably less than the hundreds of algal polysaccharide glycoside hydrolases, lyases, esterases (carbohydrate-active enzymes or CAZymes) and auxiliary enzymes that have been characterised in the context of algal polysaccharide enzymology. Furthermore, the high degree of specificity required of S1 family sulfatases results in rather limited promiscuity between sulphated algal polysaccharides even within the same family of algal polysaccharide.

**Table 1:** Table of reported algal polysaccharide-specific sulfatases from bacteria.

Name	Family	Organism	Substrate	Activity	PDB ID
4S-iota-carrageenan sulfatase (Préchoux et al., 2013)	S1_19	*P. atlantica* T6c	ι-carrageenan	*endo*-4-sulfo-D-galactose sulfatase	–
*Psc* ι-CgsA (Genicot et al., 2014)	S1_NC	*P. carrageenovora strain PscT*	ι-carrageenan	*endo*-4-sulfo-D-galactose sulfatase	–
cgsA (Ficko-Blean et al., 2017)	S1_19	*Z. galactanivorans*	ι-carrageenan	4-sulfo-D-galactose sulfatase	–
PfS1_NC (Hettle et al., 2019)	S1_NC	*P. fuliginea*	ι-carrageenan	*exo*-2-sulfo-3,6-D-anhydro-galactose sulfatase	6PT4, 6PT6, 6PT9, 6PTK
PfS1_19A (Hettle et al., 2018, 2019)	S1_19	*P. fuliginea*	ι-carrageenan κ-carrageenan	*endo*-4-sulfo-D-galactose sulfatase	6BIA, 6B0K, 6B0J, 6B1V
cgsC (Ficko-Blean et al., 2017)	S1_17	*Z. galactanivorans*	α-carrageenan	2-sulfo-3,6-D-anhydro-galactose sulfatase	–
cgsB (Ficko-Blean et al., 2017)	S1_7	*Z. galactanivorans*	κ-carrageenan	4-sulfo-D-galactose sulfatase	–
PfS1_19B (Hettle et al., 2019)	S1_19	*P.fuliginea*	κ-carrageenan	*exo*-4-sulfo-D-galactose sulfatase	6PRM, 6PSM, 6PSO
Psc κ-Cgs (Weigl and Yaphe, 1966)	NC	*P. carrageenovora* 9^T^	κ-carrageenan	*exo*-4-sulfo-D-galactose sulfatase	–
Q15XH1 (Préchoux et al., 2016)	NC	*P. atlantica* T6c	κ-carrageenan	*endo*-4-sulfo-D-galactose sulfatase	–
Q15XG7 (Préchoux et al., 2016)	NC	*P. atlantica* T6c	κ-carrageenan	*endo*-4-sulfo-D-galactose sulfatase	–
P36_S1_25 (Reisky et al., 2019)	S1_25	*F. agariphila KMM 3901*	Ulvan	*exo*-3-sulfo-L-rhamnose sulfatase	6HR5
P18_S1_7 (Reisky et al., 2019)	S1_7	*F. agariphila KMM 3901*	Ulvan	*endo*-2-sulfo-D-xylose sulfatase	6HHM
P32_S1_8 (Reisky et al., 2019)	S1_8	*F. agariphila KMM 3901*	Ulvan	*exo*-2-sulfo-D-xylose sulfatase	
SWF1 (Silchenko et al., 2018)	S1_17	*W.fucanilytica CZ1127*	Sulphated fuco-oligosaccharides	*exo*-2-sulfo-L-fucose sulfatase	–
SWF4 (Silchenko et al., 2018)	S1_25	*W. fucanilytica CZ1127*	Sulphated fuco-oligosaccharides	*exo*-3-sulfo-L-fucose sulfatase	
SWF5 (Silchenko et al., 2021)	S1_22	*W. fucanilytica CZ1127*	Sulphated fuco-oligosaccharides	*endo*-4-sulfo-L-fucose sulfatase	
PsFucS1 (Mikkelsen et al., 2021)	S1_13	*Pseudoalteromons* sp. MB47	Fucoidan		7AJ0
BpS1_11 (Robb et al., 2022)	S1_11	*B. plebeius* strain DSM 17135	Porphyran	*exo*-6-sulfo-L-galactose sulfatase	–
BuS1_11 (Robb et al., 2022)	S1_11	*B. uniformis* strain NP1^27^	Porphyran	*exo*-6-sulfo-L-galactose sulfatase	7LHA 7LJ2

NC, not classified.

Here, we review recent developments and highlight future directions for elucidating the structural and functional relationship between S1 sulfatases and their sulphated algal polysaccharide substrates. We will highlight the key role of sulfatases, not only as an integral component of marine algal polysaccharide degradation, but also as potential biocatalysts for the production of a variety of novel tailor made sulphated oligomers, which are useful products in, for example pharmaceutical or cosmetic applications.

## S1 Family Sulfatases

**Figure 1:**
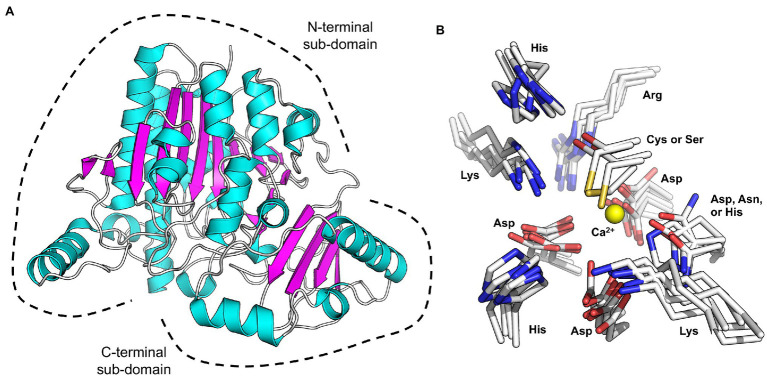
X-ray crystal structure showing the canonical sulfatase family S1 structure. **(A)** Representative marine sulfatase (PfS1_19A PDB ID: 6BIA; Hettle et al., 2018) characterised by two sub-domains of mixed α/β topology (α-helices coloured purple; β-sheets coloured cyan). Dashed line designates the secondary structure motifs comprising the N- and C-terminal sub-domains. **(B)** Structural overlay of the reported marine carbohydrate-specific sulfatase structures, of marine origin, from Table 1 highlighting the conserved residues comprising the sulfatase signature sequence motif. Only the calcium ion from the PfS1_19A structure is represented, shown as a yellow sphere.

The S1 family of sulfatases is the largest and most functionally diverse family that catalyses sulphate group elimination *via* a hydrolytic mechanism (Barbeyron et al., 2016a). To date, there are over 37,000 entrants in this family, accounting for almost 90% of all sulfatase entries in the SulfAtlas Database (Barbeyron et al., 2016a). All but two of the 18 characterised marine carbohydrate-specific sulfatases belong to this family; therefore, the S1 sulfatase family will be the focus of this review. Members of this family are Cα-formylglycine-dependent (Fgly-dependent) enzymes, in which one of the either a cysteine (Cys) or serine (Ser) residue undergoes post-translational conversion into the catalytically active Cα-Fgly residue (Hanson et al., 2004). All S1 sulfatases contain the archetypical two-domain S1 sulfatase fold. A large N-terminal domain comprises an alkaline phosphatase fold with an α/β/γ topology. Whereas, the smaller C-terminal domain contains a four stranded anti-parallel β-sheet held tightly packed against the N-terminal domain by α-helices (Hettle et al., 2018, 2019; Reisky et al., 2019; Robb et al., 2022; Figure 1A). The high degree of structural similarity in S1 sulfatases is revealed by root-mean-square-deviation values that range from ~1.2 to ~2.3 Å.

## Sulfatase Binding Site Nomenclature Defined by Recognition of the Leaving Group

Considerable effort has recently gone into trying to understand the depolymerisation of sulphate containing algal polysaccharides. Of the recently characterised marine carbohydrate-specific sulfatases, only seven with confirmed biological activity have been structurally characterised and four of these have been determined with substrate bound in the active site (Hettle et al., 2018, 2019; Silchenko et al., 2018; Mikkelsen et al., 2021; Robb et al., 2022).

These six Fgly-dependent marine S1 sulfatases, either cys-type or ser-type, possess ten highly conserved polar residues involved in sulphate recognition and sulphate ester hydrolysis. These residues consist of the cys or ser proto-catalytic residue; four residues involved in metal coordination, two of which stabilise the Fgly; and three involved in sulphate recognition and activation (Figure 1B). Of these, histidine is always found filling the role of catalytic acid and catalytic base. Some of these conserved residues are found within a 12 amino acid sequence (C/S-X-P-X-R-X-X-X-L/X-T/X-G/X-R/X), of which the first five residues (C/S-X-P-X-R) comprise the easily identifiable S1 sulfatase signature pentapeptide motif (Dierks et al., 1999). This motif defines the location of the active site for sulphate ester hydrolysis.

**Figure 2:**
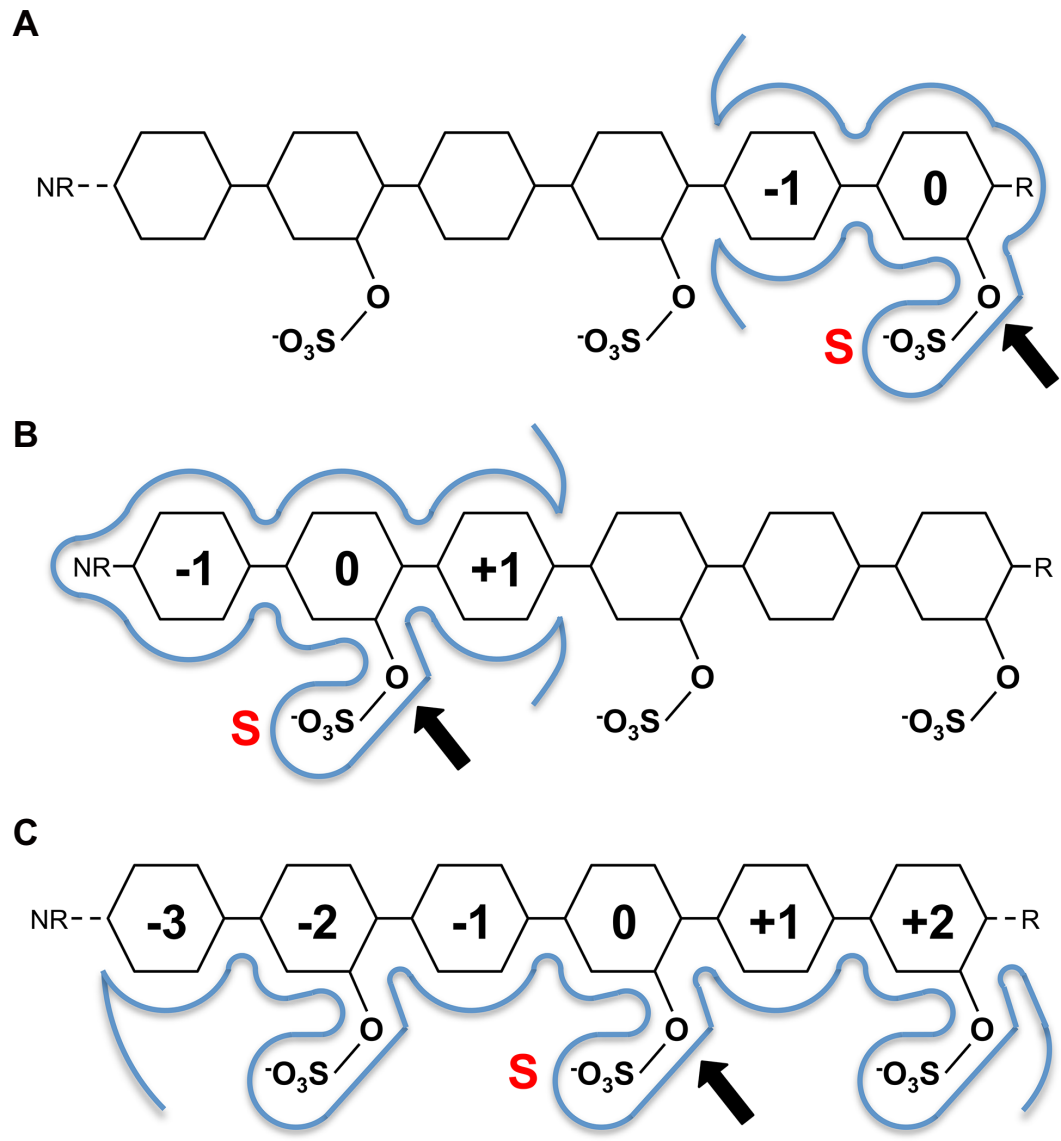
Sugar binding subsite nomenclature carbohydrate-active sulfatases as adapted from Hettle et al. (2018). Examples shown for hypothetical reducing end *exo*-acting sulfatase **(A)**, non-reducing end *exo*-sulfatase **(B)** and *endo*-acting sulfatase **(C)**. The ‘S’ subsite is indicated by a red S with a black arrow identifying the targeted sulphate ester for hydrolysis. The 0-subsite accommodates the sugar monosaccharide with the targeted sulphate ester, additional subsites are numbered negative towards the non-reducing end (NR) and positive towards the reducing end (R). Sugar residues are represented as hexagons.

This conservation of residues involved in sulphate ester hydrolysis has highlighted that the catalytic machinery utilised to recognise and hydrolyse the sulphate ester is not involved in discriminating between specific sulphated biomolecules. Rather, it is the recognition of the leaving group that confers S1 sulfatase specificity, which underpinned the carbohydrate-specific sulfatase subsite nomenclature proposed by Hettle et al. (2018). Carbohydrate-specific sulfatases can work *via* either an *exo*-hydrolytic or *endo*-hydrolytic mode of action (Helbert, 2017; Hettle et al., 2018). *Exo*-hydrolytic sulfatases catalyse the cleavage of a sulphate group from either the non-reducing or reducing end of the carbohydrate, which can be a monosaccharide, oligosaccharide or polysaccharide (Figures 2A,B). *Endo*-hydrolytic sulfatases hydrolyse sulphate esters located along the internal regions of polysaccharides and/or oligosaccharides (Figure 2C). With this in mind, the subsite nomenclature is centred around the ‘S-subsite’ which accommodates the targeted sulphate ester. Using this location as a reference, the carbohydrate moiety bearing the targeted sulphate ester is the 0-subsite. Any subsites towards the non-reducing end are numbered with increasingly negative numbers, and any towards the reducing end are numbered with increasingly positive numbers (Hettle et al., 2018; Figures 2A–C). This nomenclature can be utilised for *exo*-acting sulfatases that specifically interact with either the reducing end or the non-reducing end (Figures 2A,B), as well as *endo*-acting sulfatases that remove the sulphate group from an internal moiety of the polymer (Figure 2C).

## Understanding the Specific Role of Sulfatases in Enzymatic Cascades

From the currently characterised algal polysaccharide-specific sulfatases, it has been possible to derive a great deal regarding the molecular details underlying the structures and functions of these enzymes. In this section, we will describe the role of characterised sulfatases and their importance in carrageenan, ulvan, fucoidan and porphyran catabolism.

### A Carrageenan Perspective

**Figure 3:**
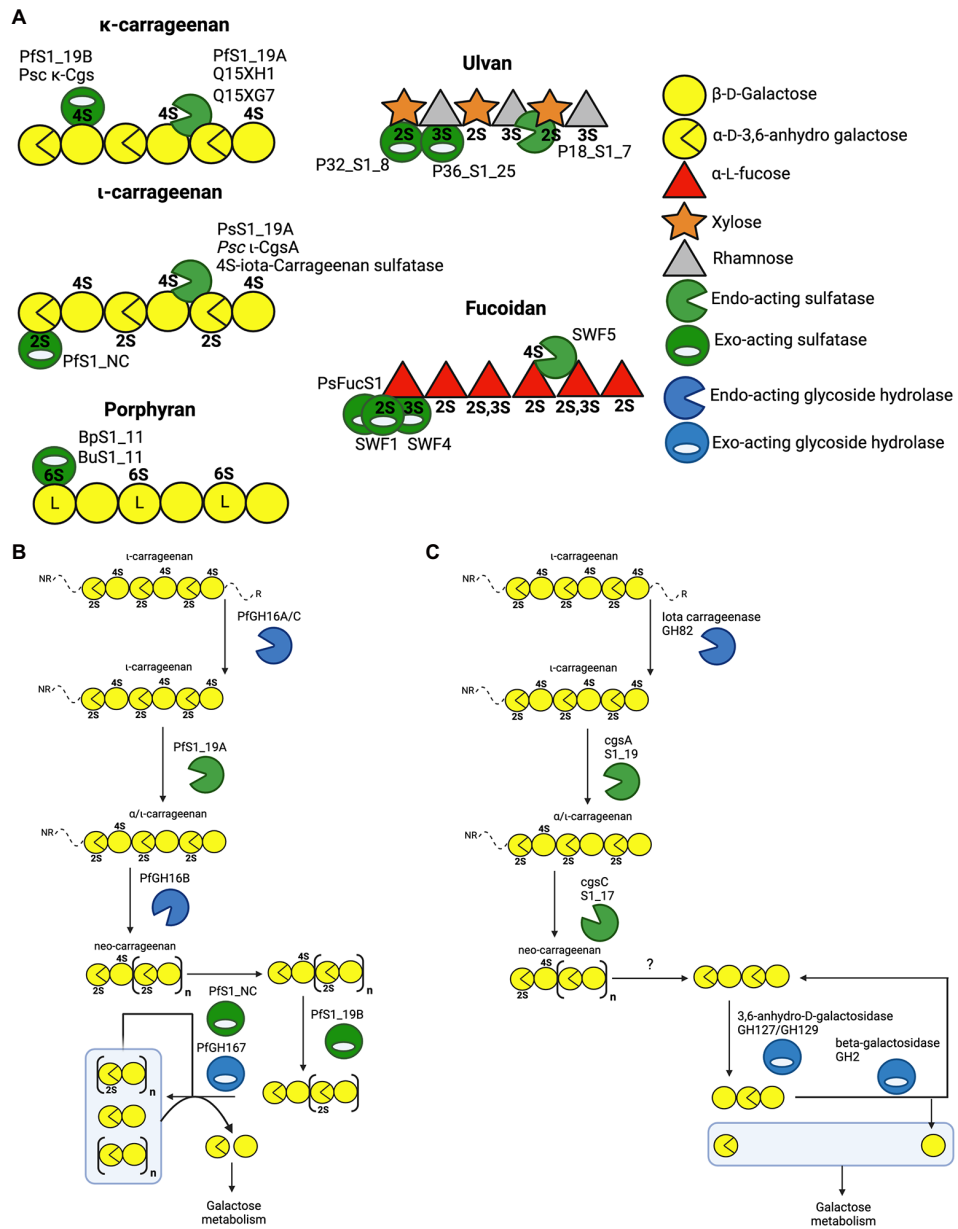
Schematics of characterised sulfatases, substrates and enzyme cascades. **(A)** Schematic of characterised marine polysaccharide sulfatases and their substrates where preferred substrate and mode of action are confirmed. The iota and kappa classes of carrageenan are provided along with porphyran as well as the highest sulphation state of fucoidan and ulvan. Characterised sulfatases are positioned at the specific sulphate ester that they have been shown to hydrolyse. **(B)** Enzyme cascade for ɩ-carrageenan catabolism in *Pseudoalteromonas fuliginea* PS47. **(C)** Enzyme cascade for ɩ-carrageenan catabolism in *Zobellia galactanivorans.*

Carrageenans are a diverse group of linear sulphated galactans produced by red algae. The carrageenan types are dependent on the presence or absence of 3,6-anhydro-D-galactose (DA) units, and the degree of free hydroxyl group sulphation at C2, C4 and C6 (2S, 4S and 6S, respectively). The most common types of carrageenan are kappa(κ)-carrageenan, iota(ι)-carrageenan and lambda(λ)-carrageenan (Knutsen et al., 1994; Usov, 1998, 2011). The carrageenan depolymerisation cascade has been studied in detail for the marine microbes *Zobellia galactanivorans* and *Pseudoalteromonas fuliginea* PS47 (Ficko-Blean et al., 2017; Hettle et al., 2019). The latter study of *P. fuliginea* employed extensive structural studies of PfS1_19A, PfS1_19B and PfS1_NC, providing unique molecular insight into the role that these enzymes play in carrageenan assimilation. Dissecting this cascade began with revealing the molecular details of how PfS1_19A hydrolyses the 4S sulphate from both ι-carrageenan and κ-carrageenan *via* an *endo*-hydrolytic mode of action to produce α/ι-carrageenan (Hettle et al., 2018; Figures 3A,B). Although PsS1_19A can act on both carrageenans it preferentially hydrolyses the 4S from ι-carrageenan. The α/ι-carrageenan is subsequently depolymerised and further de-sulphated by a series of downstream glycoside hydrolases and sulfatases, including the *exo*-acting PfS1_NC and PfS1_19B, which remove the remaining 2S and 4S, respectively (Figures 3A,B; Hettle et al., 2018, 2019). A similar cascade has been identified in *Zobellia galactanivorans* (Figure 3C; Ficko-Blean et al., 2017). In this microbe, PfS1_19A is substituted by the closely related CgsA that also produces α/ι-carrageenan; however, this product is then proposed to be further de-sulphated by CgsC, a sulfatase that removes 2S sulphate esters located along the internal regions of the oligosaccharides (Figure 3C). This produces a neo-carrageenan that can then be processed by subsequent glycoside hydrolases and likely at least two more yet to be determined *exo*-acting sulfatases. The identification of these pathways have led to the discovery of similar enzymes in other marine bacteria, or at least the placement of previously described sulfatases into these now well described pathways. The *endo*-acting sulfatases *Psc* ι-CgsA from *P. carrageenovora* 9^T^ (Genicot et al., 2014) and 4S-ι-carrageenan sulfatase from *P. atlantica* (Préchoux et al., 2013) hydrolyse the 4S sulphate from ι-carrageenan, similar to PfS1_19A and CgsA (Figure 3A). Additionally, the sulfatases CgsB from *Zobellia galactanivorans* (Ficko-Blean et al., 2017), Psc κ-Cgs from *P. carrageenovora* 9^T^ (Weigl and Yaphe, 1966), Q15Xh1 and Q15XG7 from *Pseudoalteromonas atlantica* T6c (Préchoux et al., 2016) all remove 4S from κ-carrageenan.

**Figure 4:**
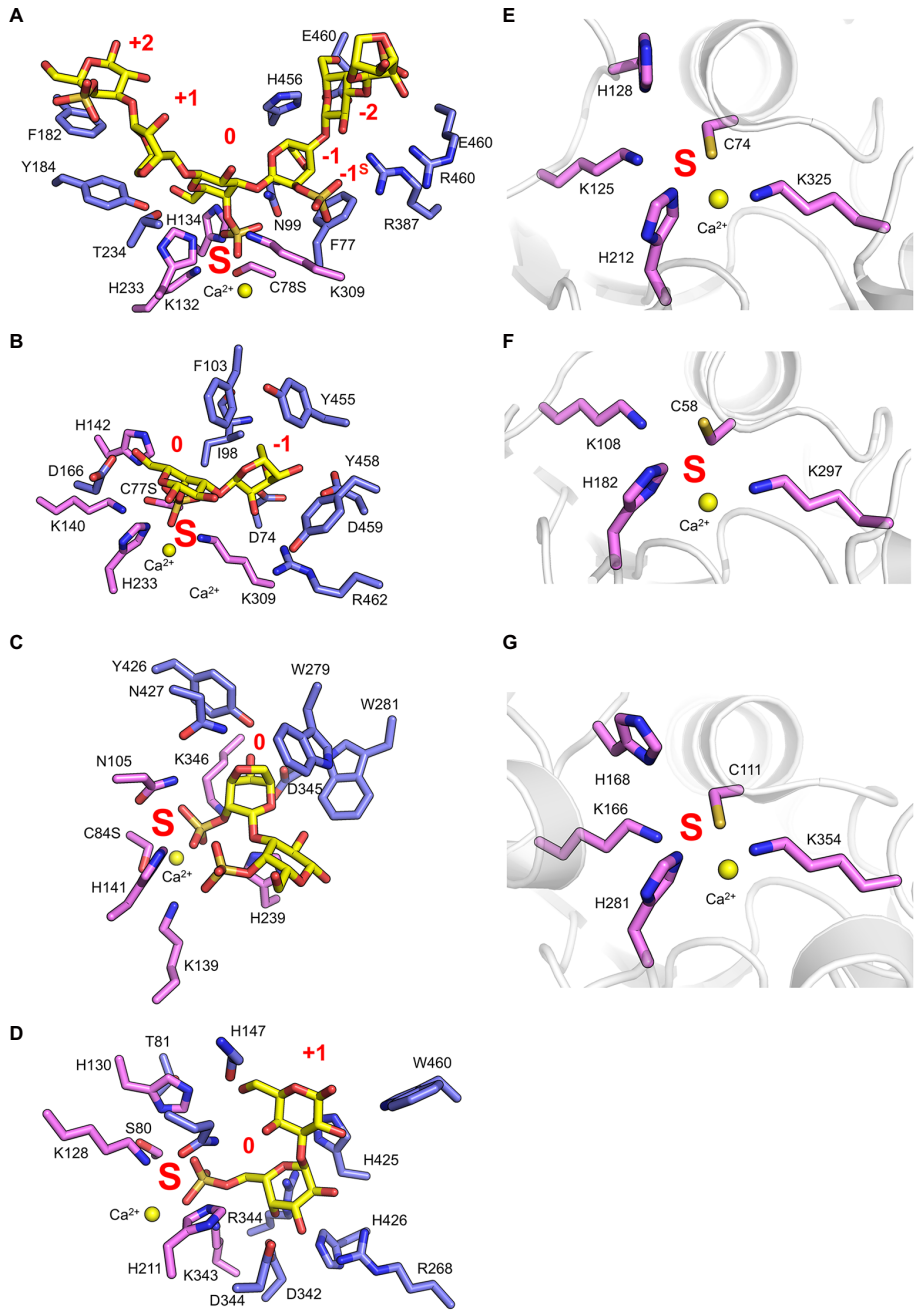
Marine carbohydrate-specific sulfatases in complex with their biological substrates. The X-ray crystal structure of PfS1_19A C78S in complex with a κ-ι-κ-carrageenan hexasaccharide [PDB ID: 6B0J, Hettle et al. (2018)] **(A)**, PfS1_19B C77S in complex with a κ-carrageenan disaccharide [PDB ID: 6PSM, Hettle et al. (2019)] **(B)**, PfS1_NC C84S in complex with an ι-carrageenan disaccharide [PDB ID: 6PT6, Hettle et al. (2019)] **(C)**, BuS1_11 S80 in complex with neoporphyrabiose [PDB ID: 7LJ2, Robb et al. (2022)] **(D)**, P18_S1_7 [PDB ID: 6HHM, Reisky et al. (2019)] **(E)**, P36_S1_25 [PDB ID: 6HR5, Reisky et al. (2019)] **(F)** and PsFuc1 [PDB ID: 7AJ0, Mikkelsen et al. (2021)] **(G)**. In all panels, S-subsite residues are shown in pink sticks, integer-subsites as purple sticks, the calcium ion is represented as a yellow sphere, and the subsites are labelled in red. In panels **(A–C)** oligosaccharides as yellow sticks, and in panels **(D–F)** the peptide backbone is shown in grey cartoon representation.

Recent work has provided insight regarding the defining structural features that confer both *endo*-hydrolytic and *exo*-hydrolytic modes of action for carrageenan sulfatases (Figure 3A). The structure of *endo*-acting PfS1_19A in complex with a κ-ι-κ-neocarrahexaose oligosaccharide highlights the extensive number of residues involved in recognition and stabilisation of internal regions of polysaccharides. These interactions occur through many classical CH/π interactions as well as non-classical interactions with unique algal residues like the DA unit in the −1 subsite (Figure 4A). In contrast, an *exo*-mode of action involves specific recognition of either the reducing or non-reducing end of the carbohydrate. PfS1_19B interacts with non-reducing end of a κ-carrageenan disaccharide confirming its designation as an *exo*-acting sulfatase. However, the S-subsite is not on the terminal residue, rather, the 0-subsite is found one residue in from the non-reducing end (Figure 4B). Furthermore, the *exo*-acting PfS1_NC (Hettle et al., 2019) from *P. fuliginea* PS47 (found in the same metabolic pathway as PfS1_19A and PfS1_19B) hydrolyses the 2S sulphate from ι-carrageenan, as does CgsC from *Z. galactanivorans* (Ficko-Blean et al., 2017). As with PfS1_19B, PfS1_NC does fully surround the non-reducing end sugar, supporting the *exo*-acting designation (Figure 4C). Here, the residue occupying the 0-subsite is at the terminus of the oligosaccharide unlike what is observed with PfS1_19B. Both PfS1_19B and PfS1_NC utilise non-classical protein-carbohydrate interactions between the unique DA unit found in carrageenans and aromatic amino acid sidechains in the active sites (Figures 4B,C).

## Characterised Sulfatases Active on Porphyran, Ulvan and Fucoidan

The number of reported sulfatases active on algal polysaccharides other than carrageenan is more limited. A recent study has characterised the *exo*-acting enzymes BpS1_11 and BuS1_11 from *Bacteroides plebeius* strain DSM 17135 and *Bacteroides uniformis* strain NP1 (28), respectively. These are human gut bacteria that have acquired algal galactan-specific polysaccharide utilisation loci (PUL), likely *via* horizontal gene transfer from marine bacteria (Robb et al., 2022). Both sulfatases are functionally equivalent and remove 6S from L-galactose-6-sulfate (L6S) at the non-reducing end of porphyran (Figure 3A). The L6S residue of porphyran is another sulphated carbohydrate residue unique to the marine environment. The binding of this residue by BuS1_11 is also distinctive of unique interactions as the L6S residue bound deep in the active site completely excludes water allowing for regioselectivity of the non-reducing end L6S and no other possible residue indicating an *exo*-mode of action (Figure 4D). This desulphation must occur prior to subsequent depolymerisation by exo-acting CAZymes for complete porphyran deconstruction. Neither enzyme was able to hydrolyse the artificial substrate 4-nitrophenyl sulphate, indicating the necessity of the carbohydrate portion of the substrate, as seen with most characterised marine carbohydrate-specific sulfatases.

There are three reported ulvan active sulfatases. Ulvans are sulphated heteropolysaccharides from green algae. The most sulphated form occurs with a repeating disaccharide unit in which a D-xylose-2-sulfate is α-1,4-linked to L-rhamnose 3-sulphate (Kidgell et al., 2019). Recently characterised ulvan sulfatases include the *exo*-acting P36_S1_25 from *F. agariphila KMM 3901* that hydrolyses 3S from rhamnose monosaccharides (Reisky et al., 2019). There is also the *endo*-acting P18_S1_7 and the *exo*-acting P32_S1_8 that both hydrolyse 2S from xylose monosaccharides, also from the same organism (Figure 3A; Reisky et al., 2019). These sulfatases are part of an extensive catabolic pathway that can sequentially convert ulvans into monosaccharides, potentially rendering certain algal blooms into a previously unexploited renewable bioresource. The ulvan specific sulfatases are yet to be reported in complex with their biological substrate; however, the apo-enzyme structures were determined for both P36_S1_25 and P18_S1_7. Both have the archetypal S1 fold and the S-subsite residues involved in sulphate coordination and sulphate ester hydrolysis (Figures 4E,F).

There are four sulfatases that are reported to have activity on fucoidans and two more potentially involved in fucoidan processing. Fucoidans are branched and highly heterogeneous sulphated fucans from brown macro algae. They have a semiregular repeating disaccharide unit of α-L-fucose-2-sulphate and α-L-fucose-2,3-sulphate with either α-1,3 or α-1,4 glycosidic linkages (Chevolot et al., 2001; Vishchuk et al., 2011). Increased structural differentiation is imparted by varying degrees of modifications including increased sulphation (2S, 3S and 4S; Chevolot et al., 2001). Biochemically characterised fucoidan sulfatases include the *exo*-acting 2S SWF1 and 3S SWF4, as well as the *endo*-acting 4S SWF5, all from *W. fucanilytica CZ1127* (Silchenko et al., 2018; Wang et al., 2019; Figure 3A). SWF5 shows promise as a biocatalyst for sulphation control on fucoidans as it can be used to directly alter the anticancer properties of fucoidans. Additionally, there are two putative fucoidan sulfatases that have been identified, but not biochemically characterised, from *Verrucomicrobium ‘Lentimonas’* sp. *CC4* (Sichert et al., 2020). These studies reveal the complexity of fucoidan catabolism and underscores why these marine glycans are probably generally recalcitrant to depolymerisation and are only slowly degraded by highly specialised organisms.

There have recently been five sulfatases isolated from *Pseudoalteromonas* sp. MB47. Through amino acid sequence comparisons using the SulfAtlas Database, of these putative sulfatases, PsSUL1, 3, 4 and PsFucS1 belong to family S1_13 while PsSUL2 belongs to S1_4 (Mikkelsen et al., 2021). PsFucS1 is the only one of these enzymes to have demonstrated sulfatase activity on fucoidan and its x-ray crystal structure has been solved revealing the canonical S1 family sulfatase fold including the S-subsite found in the active site pocket (Figure 4G; Mikkelsen et al., 2021).

Collectively, these findings highlight the specific and critical role of both *endo*-acting and *exo*-acting sulfatases in enzymatic algal polysaccharides processing. Sulfatases often play an integral role in the enzymatic cascade of depolymerizing these algal polysaccharides into monosaccharides for metabolism. As seen in the pathways utilised by *P. fuliginea* PS47 and *Z. galactivorans* (Figures 3B,C, respectively), sulfatases are found in key enzymatic steps. In some cases, these steps are critical keystone steps, such as PfS1_NC removing the non-reducing end 2S from ι-carrageenan oligosaccharides (Figures 3A,B), which is the first step in the sequential depolymerisation of ι-carrageenan from the non-reducing end. Without this step, *P. fuliginea* PS47 would not be able to utilise this agal polysaccharide as a nutrient source. All the sulfatases discussed here are highly specific, with both the degree of sulphation and composition of the polysaccharide backbone being critical factors for substrate recognition and catalytic efficiency. By specifically altering the sulphation state of polysaccharides prior to depolymerisation by subsequent CAZymes, sulfatases provide bacteria with a distinct solution to utilise these abundant polysaccharides as a nutrient source.

## The C-Terminal Sub-Domain of Family S1 Marine Carbohydrate-Specific Sulfatases Plays a Detailed Role in Substrate Recognition

**Figure 5:**
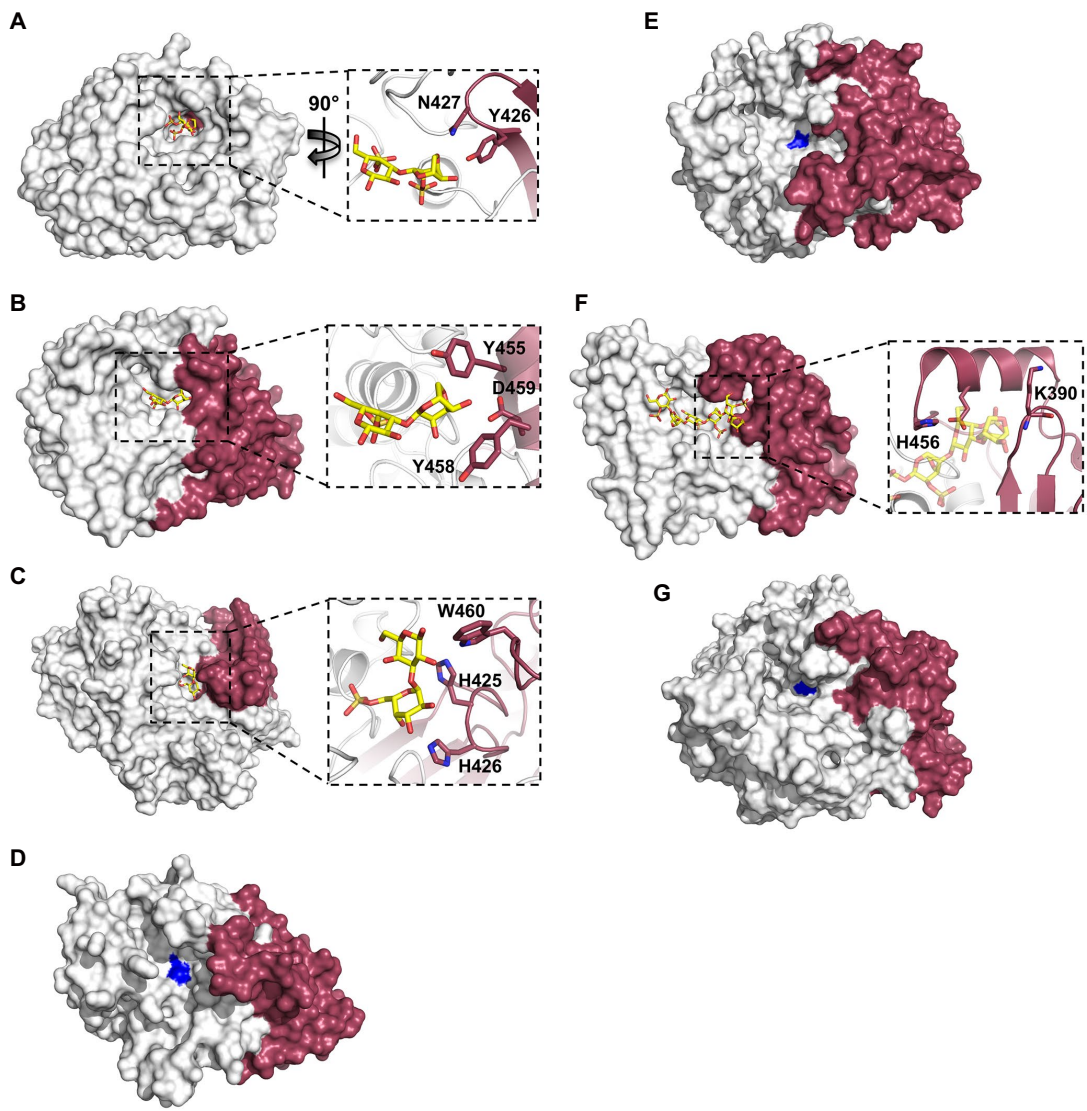
X-ray crystal structures of marine carbohydrate-specific sulfatases highlighting the importance of the C-terminal sub-domain. The X-ray crystal structure showing the solvent accessible surface of PfS1_NC C84S in complex with an ι-carrageenan disaccharide [PDB ID: 6PT6, Hettle et al. (2019)] **(A)**, PfS1_19BC77S in complex with a κ-carrageenan disaccharide [PDB ID: 6PSM, Hettle et al. (2019)] **(B)**, BuS1_11 S80 in complex with neoporphyrabiose [PDB ID: 7LJ2, Robb et al. (2022)] **(C)**, P36_S1_25 [PDB ID: 6HR5, Reisky et al. (2019)] **(D)**, PsFuc1 [PDB ID: 7AJ0, Mikkelsen et al. (2021)] **(E)**, PsS1_19A C78S in complex with a κ-ι-κ-carrageenan hexasaccharide [PDB ID: 6B0J, Hettle et al. (2018)] **(F)** and P18_S1_7 [PDB ID: 6HHM, Reisky et al. (2019)] **(G)**. In all panels, the N-terminal sub-domain is coloured white, the C-terminal sub-domain is coloured ruby. In panels **(A–F)** inset, oligosaccharides coloured yellow sticks, sulfatases are shown in cartoon representation with the same colouring as the solvent accessible surface, C-terminal sub-domain residues involved in binding are shown as ruby sticks. In panels **(D)**, **(E)**, **(G)**, the site of the catalytic Fgly is coloured blue.

The recently obtained structures of marine carbohydrate-specific sulfatases in complex with relevant substrates have highlighted very specific molecular interactions with carbohydrate components that occupy subsites outside of the highly conserved S-subsite (Hettle et al., 2018, 2019; Robb et al., 2022). Earlier structural studies of wild type apo-sulfatase structures highlighted the two distinct domains as well as the structural motifs contributing to these domains (Boltes et al., 2001; Hanson et al., 2004). These studies identified that the larger N-terminal domain aids in positioning the catalytic machinery and conserved polar residues near the base of the catalytic pocket. The role of the smaller C-terminal domain in substrate recognition remained highly speculative. Investigating the residues involved in substrate recognition by PfS1_NC, PfS1_19B and BuS1_11 reveals that the C-terminal domain contributes only a few molecular interactions involved in substrate recognition, but these few interactions are potentially critical for specificity and substrate turnover. In the case of PfS1_NC, almost the entire catalytic pocket for the recognition of ι-carrageenan is composed of N-terminal sub-domain residues apart from two (Figure 5A). The two residues contributed to the active site by the C-terminal domain are situated at the very bottom of the active site pocket and make interactions with the substrate. PfS1_19B also contains C-terminal domain residues that are crucial for the substrate recognition of κ-carrageenan di- and tetrasaccharides through the coordination of the non-reducing end DA unit in the −1 subsite (Figure 5B). The residues H425, H426 and W460 from the C-terminal sub-domain of BuS1_11 make several non-classical CH/π interactions participating in recognition at the 0 and + 1 subsites (Figure 5C). The ulvan specific *exo*-acting P36_S1_7 sulfatase requires the determination of structures in complex with substrate to confirm the contributions from the C-terminal domain for substrate recognition; however, the apo structure shows the proximity of the C-terminal domain to the active site pocket, suggesting its role in substrate recognition (Figure 5D). Like the ulvan specific sulfatases, PsFucS1 structure also reveals a potential contribution from the C-terminal domain to the active site pocket (Figure 5E).

The contribution of C-terminal domain residues from the *endo*-acting PfS1_19A sulfatase in complex with a κ-ι-κ-carrageenan hexasaccharide indicates that participation of the C-terminal domain to substrate recognition is not exclusive to *exo*-acting sulfatases. The recognition of residues in the negative subsites is largely composed of C-terminal residues (Figure 5F). This open C-terminal domain architecture in the *endo*-acting carrageenan sulfatase is what allows for the accommodation of an extended carbohydrate substrate. This appears to hold true for other *endo*-acting marine carbohydrate-specific sulfatases. Like PfS1_19A, in the *endo*-acting P18_S1_7 apo structure, the C-terminal domain appears to form one of the walls of the active site groove (Figure 5G).

Overall, the structural analyses of sulfatases that are active on algal polysaccharides point to a key role of the C-terminal domain in substrate recognition. Further, we suggest that this may not be exclusive to this particular class of S1 sulfatases but may be relevant to the S1 family as a whole. However, more structures of sulfatases that are active on algal polysaccharides (or other sulphated carbohydrates) are needed to inform potential predictions of a role substrate recognition.

## Application and Future Directions

Algal polysaccharides represent an extraordinary natural source of chemical diversity. Like polysaccharides from land plants, they have huge variation in monosaccharide composition, degree of polymerisation and branching. Algal polysaccharides have additional modifications, such as sulphate ester substitutions, that impart further chemical diversity. The unique chemistry of these natural polymers confers a range of functional properties, such as bioactivities (antimicrobial and immune-modulation) and gelling properties, the latter of which is important for food industries (Jiao et al., 2011; Vishchuk et al., 2011; Cunha and Grenha, 2016; Wang et al., 2019; Kidgell et al., 2020; Kwon et al., 2020; Silchenko et al., 2021). Currently lacking are tools to specifically determine chemical details of these natural polymers and tailor this chemistry in order to increase commercial potential. This is one factor that is hindering the large-scale commercialisation of algal polysaccharides for certain applications. Marine carbohydrate-specific sulfatases found in macroalgae utilising bacteria are potential tools for this job. Current chemical techniques cannot match the incredible specificity, speed and cost-effective way these enzymes catalyse chemical reactions required to industrially manipulate algal polysaccharides for commercialisation.

There are several reports and review articles covering the broad range of bioactive properties possessed by algal polysaccharides (Jiao et al., 2011; Vishchuk et al., 2011; Cunha and Grenha, 2016; Wang et al., 2019; Kidgell et al., 2020; Kwon et al., 2020; Silchenko et al., 2021). Application of ulvans in the food and pharmaceutical industry are less advanced than that of carrageenans and fucoidans, yet there are reports of their potential (Kidgell et al., 2019). Carrageenans are used extensively in the food industry as gelling, stabilising and clarifying agents. Fucoidans are becoming an important source of bioactive molecules (Wang et al., 2019; Kwon et al., 2020). For example, the first new Alzheimer’s drug developed in 17 years (Wang et al., 2019) and two anti-viral drug candidates (RPI-27 and RPI-28; Kwon et al., 2020) that effectively inhibit SARS-CoV-2 are both derived from fucoidans.

**Figure 6:**
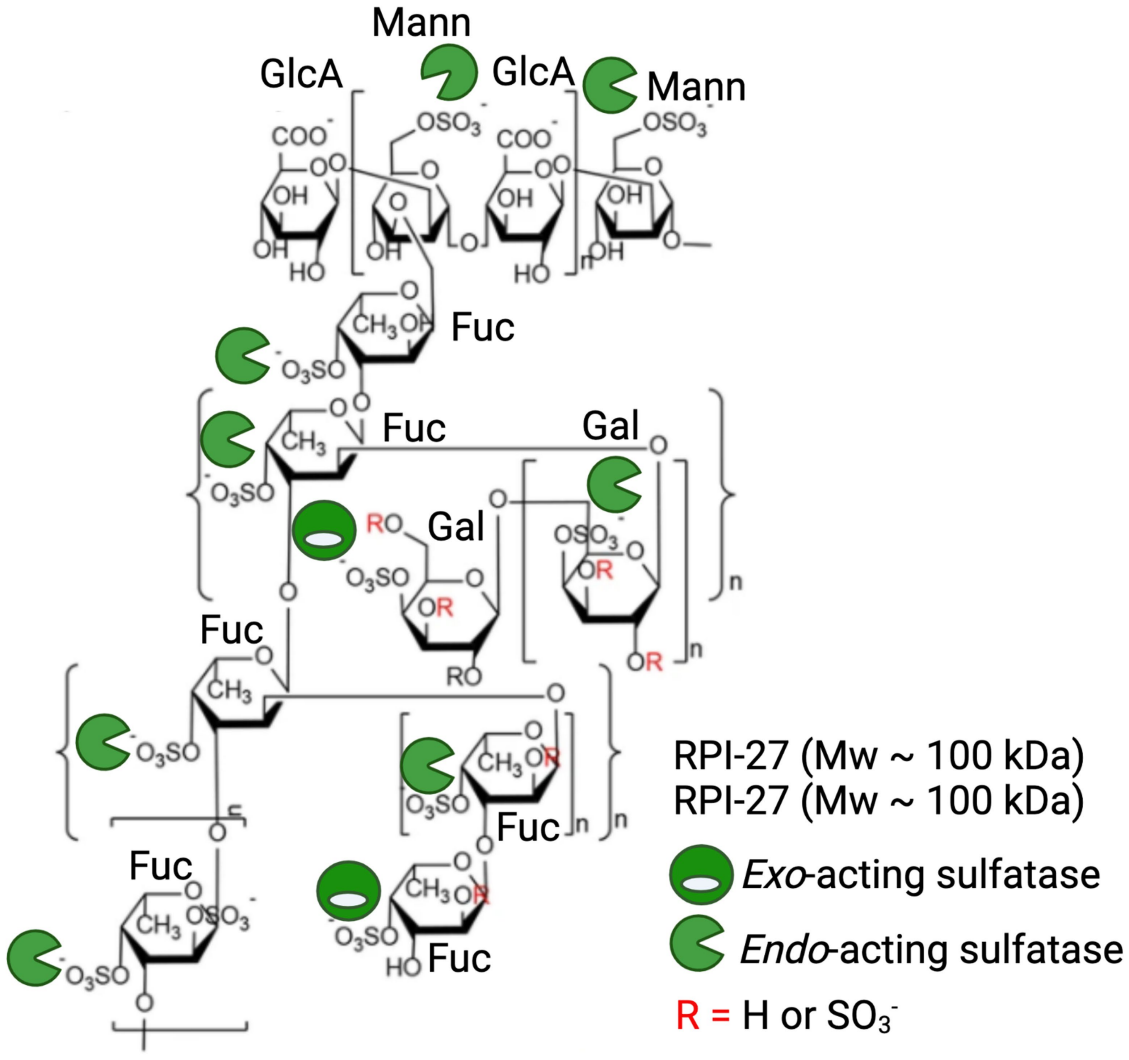
Application of marine polysaccharide sulfatases to enhance anti-viral activity of the fucoidan polysaccharides RPI-27 and RPI-28. Oligomeric chemical structure adapted from Kwon et al. (2020) and labelled with a combination of potential *endo*-acting and *exo*-acting sulfatases to specifically tailor the sulphation pattern of RPI-27 and RPI-28.

Given the detailed chemical and structural description of RPI-27 and RPI-28 and their highly relevant bioactivity, we will discuss these fucoidans as a case study in highlighting the potential of sulfatases in enhancing commercial potential of algal polysaccharides. RPI-27 is approximately 100 kDa with a high degree of branching, back bone modifications and sulphation (Figure 6; Jin et al., 2012; Kwon et al., 2020). RPI-28 has a molecular weight of approximately 12 kDa and appears to be an oligosaccharide of RPI-27. Both have a backbone containing primarily glucuronic acid, mannose, fucose and galactose. These complex molecules are highly sulphated with sulphation occurring primarily on mannose (6S), fucose (4S) and galactose (4S). Kwon et al. (2020) show that both molecules work by binding to the spike protein of virus particles and attribute the binding largely to the sulphate esters (Kwon et al., 2020).

Treatment of these molecules with a combination of specific *endo*-acting and *exo*-acting sulfatases to assess how alterations in sulphation patterns effect bioactivities in these, or similar molecules, is an exciting future direction. The repeating unit of these molecules have at least 10 sulphate esters per 12 monosaccharides, providing 10 available sites for enzymatic remodelling and subsequent alterations in potency of bioactivities. *Endo*-acting sulfatases would be the most effective at remodelling as they have the capacity to remove the most abundant sulphate esters, which occur along the polysaccharide backbone (Figure 6).

To date, the closest characterised sulfatase that may be considered a biocatalyst for remodelling these, or similar molecules, would be the 4S *endo*-acting SWF5 from *W. fucanilytica* CZ1127 which has already shown promise as a biocatalyst for sulphation control on fucoidans (Silchenko et al., 2021). Also, the five characterised 4S *endo*-acting κ-carrageenan sulfatases (PfS1_19A, PfS1_19B and PfS1_NC from *P. fuliginea* and Q15XH1 and Q15XG7 and the 4S-iota carrageenan sulfatase from *P. atlantica* T6c) have potential to remodel the 4S galactose branch of these molecules that have similar chemical structure to κ-carrageenan (Weigl and Yaphe, 1966; Préchoux et al., 2016; Ficko-Blean et al., 2017; Hettle et al., 2018, 2019).

It is likely that there is a treasure trove of novel, but yet to be discovered, marine carbohydrate-specific sulfatases encoded in the genomes of marine bacteria that hold great potential as sulphation remodelling biocatalysts. Furthermore, given the significant molecular insight from recent structural characterisation of sulfatases, it may now be possible to engineer these sulfatases to improve or alter their activities. For example, we now know how critical key residues of the C-terminal sub-domain are involved in the highly specific substrate binding of these enzymes and subsequent catalytic turnover efficiency. Given this knowledge, it is now possible to engineer these enzymes to have tailored substrate specificity, and perhaps more efficient catalysis.

These observations highlight that now is an exciting time to discover and characterise carbohydrate-specific sulfatases that are active on algal polysaccharides. More knowledge regarding the diversity and capacity of these sulfatases is essential to better understand the critical role that they play, not only in marine microbial metabolism or human gut health, but also as key biocatalysts in future polysaccharide industrial application.

## Author Contributions

All authors listed have made a substantial, direct, and intellectual contribution to the work and approved it for publication.

## Funding

The preparation of this article was supported by a Natural Sciences and Engineering Research Council of Canada Discovery Grant (FRN 04355).

## Conflict of Interest

The authors declare that the research was conducted in the absence of any commercial or financial relationships that could be construed as a potential conflict of interest.

## Publisher’s Note

All claims expressed in this article are solely those of the authors and do not necessarily represent those of their affiliated organizations, or those of the publisher, the editors and the reviewers. Any product that may be evaluated in this article, or claim that may be made by its manufacturer, is not guaranteed or endorsed by the publisher.
